# Efficacy and safety of transcatheter aortic valve replacement in aortic stenosis patients at low to moderate surgical risk: a comprehensive meta-analysis

**DOI:** 10.1186/s12872-017-0668-1

**Published:** 2017-08-24

**Authors:** Ahmed Elmaraezy, Ammar Ismail, Abdelrahman Ibrahim Abushouk, Moutaz Eltoomy, Soha Saad, Ahmed Negida, Osama Mahmoud Abdelaty, Ahmed Ramadan Abdallah, Ahmed Magdy Aboelfotoh, Hossam Mahmoud Hassan, Aya Gamal Elmaraezy, Mahmoud Morsi, Farah Althaher, Moath Althaher, Ammar M. AlSafadi

**Affiliations:** 1 Faculty of Medicine, Al-Azhar University, Cairo, Egypt; 2 NovaMed Medical Research Association, Cairo, Egypt; 3 Faculty of Medicine, Ain Shams University, Cairo, Egypt; 4 Genetic Engineering & Biotechnology Research Institute (GEBRI), University of Sadat City, Sadat City, Egypt; 5 Faculty of Medicine, Tanta University, Tanta, Egypt; 6 Faculty of Medicine, Zagazig University, Zagazig, Egypt; 7 Ahmed Maher Teaching Hospital, Cairo, Egypt; 8 Faculty of Medicine, Benha University, Benha, Egypt; 9 Faculty of Medicine, Beni Suef University, Beni suef, Egypt; 10 Faculty of Medicine, Minoufia University, Shebin El-Kom, Egypt; 11 Faculty of Medicine, Misr University for science and technology (MUST), 6th of October City, Giza, Egypt; 12 Faculty of Medicine, Damascus University, Damascus, Syria

**Keywords:** Aortic stenosis, Aortic valve replacement, Meta-analysis, Surgical, Transcatheter

## Abstract

**Background:**

Recently, transcatheter aortic valve replacement (TAVR) has become the procedure of choice in high surgical risk patients with aortic stenosis (AS). However, its value is still debated in operable AS cases. We performed this meta-analysis to compare the safety and efficacy of TAVR to surgical aortic valve replacement (SAVR) in low-to-moderate surgical risk patients with AS.

**Methods:**

A systematic search of five authentic databases retrieved 11 eligible studies (20,056 patients). Relevant Data were pooled as risk ratios (RRs) or standardized mean differences (SMD), with their 95% confidence interval, using Comprehensive Meta-Analysis and RevMan software for windows.

**Results:**

At one-year of follow-up, the pooled effect-estimates showed no significant difference between TAVR and SAVR groups in terms of all-cause mortality (RR 1.02, 95% CI [0.83, 1.26], stroke (RR 0.83, 95%CI [0.56, 1.21]), myocardial infarction (RR 0.82, 95% CI [0.57, 1.19]), and length of hospital stay (SMD -0.04, 95% CI [−0.34, 0.26]). The incidence of major bleeding (RR 0.45, 95% CI [0.24, 0.86]) and acute kidney injury (RR 0.52, 95% CI [0.30, 0.88]) was significantly lower in the TAVR group, compared to the SAVR group. However, TAVR was associated with a higher risk of permanent pacemaker implantation (RR 2.57, 95% CI [1.36, 4.86]), vascular-access complications at 1 year (RR 1.99, 95%CI [1.04, 3.80]), and paravalvular aortic regurgitation at 30 days (RR 3.90, 95% CI [1.25, 12.12]), compared to SAVR.

**Conclusions:**

Due to the comparable mortality rates in SAVR and TAVR groups and the lower risk of life-threatening complications in the TAVR group, TAVR can be an acceptable alternative to SAVR in low-to-moderate risk patients with AS. However, larger trials with longer follow-up periods are required to compare the long-term outcomes of both techniques.

**Electronic supplementary material:**

The online version of this article (doi:10.1186/s12872-017-0668-1) contains supplementary material, which is available to authorized users.

## Background

Aortic stenosis (AS) is the most prevalent valvular heart disease in the elderly [1]. An epidemiological study estimated that more than one in eight individuals over the age of 75 years has a moderate to severe AS [2]. Another meta-analysis revealed that the pooled prevalence of the disease among the elderly is 12.4% and estimated that there are more than 291,000 candidates for aortic valve replacement in North America and Europe [3].

Although surgery is still considered the intervention of choice in operable cases of severe AS, transcatheter aortic valve replacement (TAVR) is continuously gaining ground in these lower risk groups [4]. This growing trend is justified by multiple reasons including the remarkable technical advances in the valve replacement procedure which now allows for easy repositioning and removal, the minimally invasive approach that permits performing under local anesthesia [5], as well as the fact that TAVR is a common patient preference among surgically fit cases due to its shorter hospital stay, lower risk of bleeding and mild post-interventional symptoms [6].

Nevertheless, the increasing TAVR drift towards lower surgical risk strata lacks a solid ground of evidence and does not adhere to the well-established guidelines [7]. In fact, only four randomized controlled trials (RCTs) addressed this issue including the PARTNER-II, US pivotal, NOTION, and the prematurely-terminated STACATTO trial [8–11]. Given this paucity of RCTs, observational studies are rendered a legitimate strategy to assess the comparative effectiveness of both procedures in operable patients [12].

We aimed to synthesize level I evidence from published randomized trials and observational studies as to whether or not TAVR could be compared to surgery in terms of efficacy and safety outcomes in low-to-moderate surgical risk patients with AS.

## Methods

We performed this meta-analysis in accordance to the guidelines of the Cochrane handbook for systematic reviews of interventions [13] and the Preferred Reporting Items for Systematic Reviews and Meta-analyses (PRISMA statement guidelines) [14].

### Literature search strategy

We performed a comprehensive search of five authentic databases (PubMed, Scopus, Web of science, Embase, and Cochrane Central Register of Controlled Trials (CENTRAL)) using the following strategy: [Aortic Stenosis OR Aortic Valve Stenosis OR Aortic Valve Replacement OR Aortic Valve Implantation OR Heart Valve Replacement AND Transcatheter OR TAVR OR Transfemoral OR Transapical AND Surgical Aortic Valve Replacement OR SAVR OR Surgical AVR AND Low Risk OR Moderate Risk OR Intermediate Risk]. There was no restriction by the language of the study or year of publication. We screened the bibliography of eligible articles for any relevant studies and the clinical trial registry (Clinicaltrials.gov) for any ongoing or unpublished studies.

### Eligibility criteria and study selection

We included both RCTs and non-randomized studies (prospective and retrospective observational studies) if they matched the following criteria: (1) Population: Patients with severe AS and a low-to-moderate surgical risk [defined as a logistic Euroscore for cardiac operative risk evaluation (≤ 20%) or a Society of Thoracic Surgeons (STS) score below 8%], (2) Intervention: Transcatheter Aortic Valve Replacement (TAVR: through all routes including transfemoral, transapical, and transaxillary routes), (3) Comparator: Surgical Aortic Valve Replacement (SAVR), and (4) Outcomes: Studies that at least included one efficacy (mortality) or safety outcome.

We excluded case reports, case series, and studies that exclusively enrolled patients with high surgical risk. Eligibility screening was conducted in a two step-wise manner (title/abstract screening and full-text screening). Each step was conducted by three reviewers and consensus was obtained upon consulting a fourth reviewer (Abushouk AI).

### Data extraction

Three independent authors extracted the relevant data and another reviewer (Elmaraezy A) resolved disagreements. The extracted data included (1) Study year and design, (2) Baseline characteristics of enrolled patients, and (3) Outcomes including the length of hospital stay and the incidence of all-Cause mortality (efficacy outcome), major adverse cardiovascular and cerebrovascular events (MAACE), stroke, myocardial infarction (MI), major life-threatening bleeding, acute kidney injury (AKI), vascular access complications (VAC), paravalvular aortic regurgitation (AR), and permanent pacemaker implantation (PPI).

### Risk of bias assessment

Three independent reviewers used the Cochrane risk of bias tool, clearly described in (chapter 8.5) of the Cochrane handbook for systematic reviews of interventions 5.1.0 [13], to assess the risk of bias within included RCTs. For cohort and case-control studies, we used the Newcastle Ottawa scale (NOS) for detection of bias in non-randomized studies [15]. This tool assesses the risk of bias in observational studies based on reporting of three important domains: selection of the study subjects, comparability of groups regarding demographic characteristics and important potential confounders, and ascertainment of the prespecified outcome. Whenever an outcome included 10 or more studies, we assessed for publication bias, using the Egger’s test [16].

### Data synthesis

Dichotomous data for efficacy and safety outcomes were pooled as risk ratios (RRs), using the Mantel-Haenszel method. Data for hospital stay duration were pooled as a standardized mean difference (SMD), using the Inverse Variance (I-V) method. All statistical analyses in this study were performed using the Comprehensive Meta-Analysis (Biostat Inc) and RevMan (version 5.3) software for windows. Heterogeneity was assessed using the Chi-Square test and its extent was measured using the I-Square test. When a significant heterogeneity was found, the analysis was conducted under the random-effects model. In each included outcome, we performed a subgroup analysis by the endpoint of assessment (30 days, 1, 2, or 3 years after the procedure).

## Results

### Literature search results

**Fig. 1:**
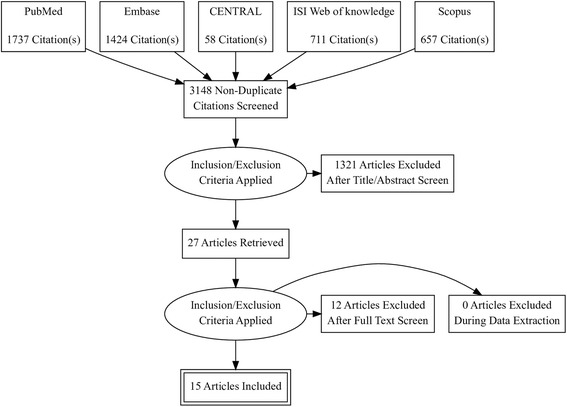
PRISMA Flow Diagram of literature search and study selection process

Our literature search retrieved 4587 studies. Of them, 27 full text articles were assessed for eligibility. Finally, 11 studies (reported in 15 published articles) [7–12, 17–25] were included in this meta-analysis [20,056 Patients]. The flow of study selection is shown in our PRISMA flow diagram (Fig. 1). Four eligible studies were RCTs, while the remaining seven studies included five prospective cohort and two retrospective studies. The summary of included studies and baseline characteristics of enrolled patients are shown in Table 1 and Table 2, respectively.

**Table 1:** shows a summary of the design and main findings of included studies

Study ID	Study Design	Population	Valve for TAVR	Follow up	Main Finding
I. Randomized Controlled Trials					
PARTNER II Trial [8]	Prospective, multicenter, randomized trials	Intermediate surgical risk patients who had senile degenerative aortic valve stenosis with Echo-derived criteria: mean gradient >40 mmHg or jet velocity greater than 4.0 m/s and an initial aortic valve area of <0.8 cm2.	Edwards SAPIEN XT	2 years	SAVR and TAVR procedures were similar in the rate of mortality and disabling stroke. However, TAVR was associated with lower rates of bleeding, AKI, and new-onset AF, while SAVR was associated with lower rates of VAC and paravalvular AR.
STACATTO Trial [11]	Prospective, randomized trial	Operable patients, older than 75 years old with sever aortic stenosis (valve area < 1 cm^2^)	Edwards SAPIEN	3 months	Despite the premature termination, the authors concluded that transapical TAVR has more complications and lower success rates than SAVR in low risk patients.
NOTION Trial [9]	Multicenter, randomized, superiority trial	Patients > 70 years old with severe aortic valve stenosis and no significant coronary artery disease	CoreValve	2 years	After 2 years of follow up, the rates of mortality, stroke, and myocardial infarction were similar in both SAVR and TAVR groups.
US PIVOTAL Trial [10]	Multicenter, randomized, non-inferiority trial	Intermediate surgical risk patients with severe aortic stenosis and an aortic valve Area ≤ 0.8 cm^2^ or an AVA index ≤0.5 cm2/m^2^	CoreValve	2 years	The rates of 2-year death or stroke were lower in the TAVR group, compared to the SAVR group.
II. Observational (Prospective and Retrospective Studies)					
OBSERVENT Study [12]	Observational, prospective, multicenter cohort study	Adult patients with a diagnosis of severe AS and low surgical risk	Edwards SAPIEN XT, CoreValve	3 years	SAVR patients are at a higher risk of blood transfusion, while TAVR patients have a higher risk of VAC, paravalvular AR, and atrioventricular block.
Osnabrugge et al. 2012 [21]	Prospective, Cohort study	Patient with severe aortic stenosis and an intermediate surgical risk	CoreValve	1 year	At 1 year follow up, the costs of TAVR were significantly higher than SAVR in intermediate risk patients owing to the higher cost of the transcatheter valve.
Piazza et al. 2013 [22]	Prospective, cohort study	Intermediate surgical risk patients with severe aortic stenosis and an aortic valvular orifice area of <1.0 cm^2^	CoreValve	1 year	In intermediate risk patients, TAVR and SAVR had comparable rates of in-hospital and 30-day mortality, with more benefit of TAVR in women
Schymik et al. 2015 [24]	Prospective, cohort study	Patients ≥75 years old with sever aortic stenosis and less than high surgical risk	Edwards SAPIEN XT, CoreValve	3 years	Both groups had comparable mortality rates. However, the rates of VAC, PPI, and AR were higher in the TAVR group and the risk of major bleeding was higher in the SAVR group.
Castrodeza et al. 2016 [18]	Prospective cohort study	Patients with severe aortic stenosis and low to intermediate surgical risk	Edwards SAPIEN XT, CoreValve	1 year	In intermediate risk patients, TAVR is feasible and has comparable mortality and stroke rates to SAVR.
Latib et al. 2012 [19]	Case control study	Intermediate surgical risk patients with severe symptomatic aortic stenosis.	Edwards SAPIEN XT, CoreValve	1 year	The rates of mortality and stroke were comparable in both groups; however, transfemoral TAVR group had a higher incidence of VAC and SAVR group had a higher incidence of AKI.
Möllmann et al. 2016 [20]	Retrospective cohort study	Patients with severe aortic stenosis at low, intermediate, and high surgical risk.	–	1 year	Compared to SAVR, transfemoral TAVR has similar in-hospital mortality in low risk patients and lower in-hospital mortality in intermediate-to-high risk patients.

***Abbreviations*** **:** *AF* Atrial fibrillation, *AKI* Acute kidney injury, *AR* Aortic regurgitation, *AVA* Aortic valve area, *PPI* Permanent pacemaker implantation, *SAVR* Surgical aortic valve replacement, *TAVR* Transcatheter aortic valve replacement, *VAC* Vascular access complications

**Table 2:** shows baseline characteristics of enrolled patients in included studies

Study ID	Arm	Demographics			Surgical Risk		Comorbidities					
		Sample Size	Age	Male sex (%)	EURO score	STS score	CAD	Previous PCI	HTN	Cerebral Vascular Disease	Peripheral Vascular Disease	Diabetes Mellitus
I. Randomized Controlled Trials												
PARTNER II trial [8]	SAVR	1021	81.7 ± 6.7	560 (54.8)	NA	5.8 ± 1.9	679 (66.5)	282 (27.6)	NA	317 (31)	336 (32.9)	349 (34.2)
	TAVR	1011	81.5 ± 6.7	548 (54.2)	NA	5.8 ± 2.1	700 (69.2)	274 (27.1)	NA	325 (32.1)	282 (27.9)	381 (37.7)
STACATTO trial [11]	SAVR	36	82 ± 4.4	12 (33.3)	10.3 ± 5.8	3.4 ± 1.2	NA	NA	NA	1 (2.8)	3 (8.3)	3 (8.3)
	TAVR-TA	34	80 ± 3.6	9 (26.5)	9.4 ± 3.9	3.1 ± 1.5	NA	NA	NA	1 (2.9)	2 (5.9)	1 (2.9)
NOTION trial [9]	SAVR	135	79 ± 4.7	71 (52.6)	8.9 ± 5.5	3.1 ± 1.7	6 (4.4)	12 (8.9)	103 (76.3)	22 (16.3)	9 (6.7)	28 (20.7)
	TAVR	145	79.2 ± 4.9	78 (53.8)	8.4 ± 4	2.9 ± 1.6	8 (5.5)	11 (7.6)	103 (71)	24 (16.6)	6 (4.1)	26 (17.9)
US PIVOTAL trial [10]	SAVR	359	83.3 ± 6.3	188 (52.4)	18.8 ± 13.2	7.5 ± 3.3	273 (76)	135 (37.6)	345 (96.1)	NA	150 (42)	162 (45.1)
	TAVR	391	83.2 ± 7.1	207 (52.9)	17.7 ± 13	7.3 ± 3.0	295 (75.4)	134 (34.3)	372 (95.1)	NA	159 (41)	136 (34.8)
II. Observational (Prospective and Retrospective Studies)												
OBSERVENT Study [12]	SAVR	1383	72 ± 9	768 (55.5)	6.1 ± 7.1	NA	NA	95 (6.8)	NA	NA	176 (12.7)	346 (25)
	TAVR-TA	602	82 ± 6	243 (40.4)	15.2 ± 14.3	NA	NA	151 (25.1)	NA	NA	114 (18.9)	145 (24.1)
	TAVR-TF	123	82 ± 7	54 (43.9)	14.7 ± 11.3	NA	NA	31 (25.2)	NA	NA	47 (38.2)	34 (27.6)
Osnabrugge et al. 2012 [21]	SAVR	42^a^	79.3 ± 5.5	22 (52.4)	12.5 ± 6.4	NA	20 (47.6)	NA	NA	2 (4.8)	4 (9.5)	8 (19)
	TAVR	42^a^	78.8 ± 6.6	21 (50)	12.9 ± 6.8	NA	20 (47.6)	NA	NA	2 (4.8)	3 (7.1)	11 (26.2)
Piazza et al. 2013 [22]	SAVR	405	79.4 ± 4.8	179 (44.1)	17.5 ± 12.1	NA	96 (57.8)	NA	286 (81.7)	30 (7.4)	41 (10.1)	98 (25.7)
	TAVR	405	79.9 ± 6	175 (43.2)	17.1 ± 10.7	NA	94 (56.6)	NA	301 (86)	40 (9.9)	33 (8.2)	111 (27.4)
Schymik et al. 2015 [24]	SAVR	216	78.2 ± 4.6	51.4%	8.8 ± 2.8	NA	48.1%	NA	NA	NA	6.9%	NA
	TAVR	216	78.3 ± 5.2	46.3%	8.7 ± 2.7	NA	48.1%	NA	NA	NA	5.1%	NA
Castrodeza et al. 2016 [18]	SAVR	70	78 ± 5.6	34 (48.6)	9.3 ± 3.9	4.3 ± 2.4	NA	NA	51 (72.9)	NA	NA	18 (25.7%)
	TAVR	70	79 ± 7.7	36 (51.4)	9.4 ± 3.8	4.6 ± 2.1	NA	NA	45 (64.3)	NA	NA	26 (37.1%)
Latib et al. 2012 [19]	SAVR	111	79.4 ± 3	49 (44.1)	24.4 ± 13.4	4.6 ± 2.6	51 (45.9)	NA	77 (69.4)	20 (18)	38 (34.2)	24 (21.6)
	TAVR	111	80.5 ± 6.9	49 (44.1)	23.2 ± 15.1	4.6 ± 2.3	44 (39.6)	NA	78 (70.3)	16 (14.4)	29 (26.1)	21 (18.9)
Möllmann et al. 2016 [20]	SAVR	9899	68.1 ± 11	60.4%	NA	NA	21.1%	8.9%	NA	5%	4.5%	24.1%
	TAVR-TF	7620	81.2 ± 6.1	44.5%	NA	NA	55%	29.6%	NA	7.8%	14.4%	33.6%
	TAVR-TA	2821	80.3 ± 6.6	52.4%	NA	NA	60.8%	30.4%	NA	9.2%	32%	33.4%

^a^Following propensity score matching. The presented data are either frequency (%) or mean ± standard deviation, unless stated otherwise

*Abbreviations*: *CAD* Coronary artery disease, *HTN* Hypertension, *NA* Not Available, *PCI* Percutaneous coronary intervention, *SAVR* Surgical aortic valve replacement, *STS* Society of Thoracic Surgeons, *TAVR* Transcatheter aortic valve replacement, *TA* Trans-Apical, *TF* Trans-Femoral

### Risk of bias in included studies

The risk of bias in included RCTs ranged from low to moderate as assessed by the Cochrane Risk of Bias tool. Authors’ judgements on the risk of bias in included RCTs are illustrated in Additional file 1. The risk of bias in included observational studies was low as assessed by the Newcastle Ottawa scale (mean = 8 out of 9 asterisks).

### Safety and efficacy outcomes

#### All-cause mortality

**Fig. 2:**
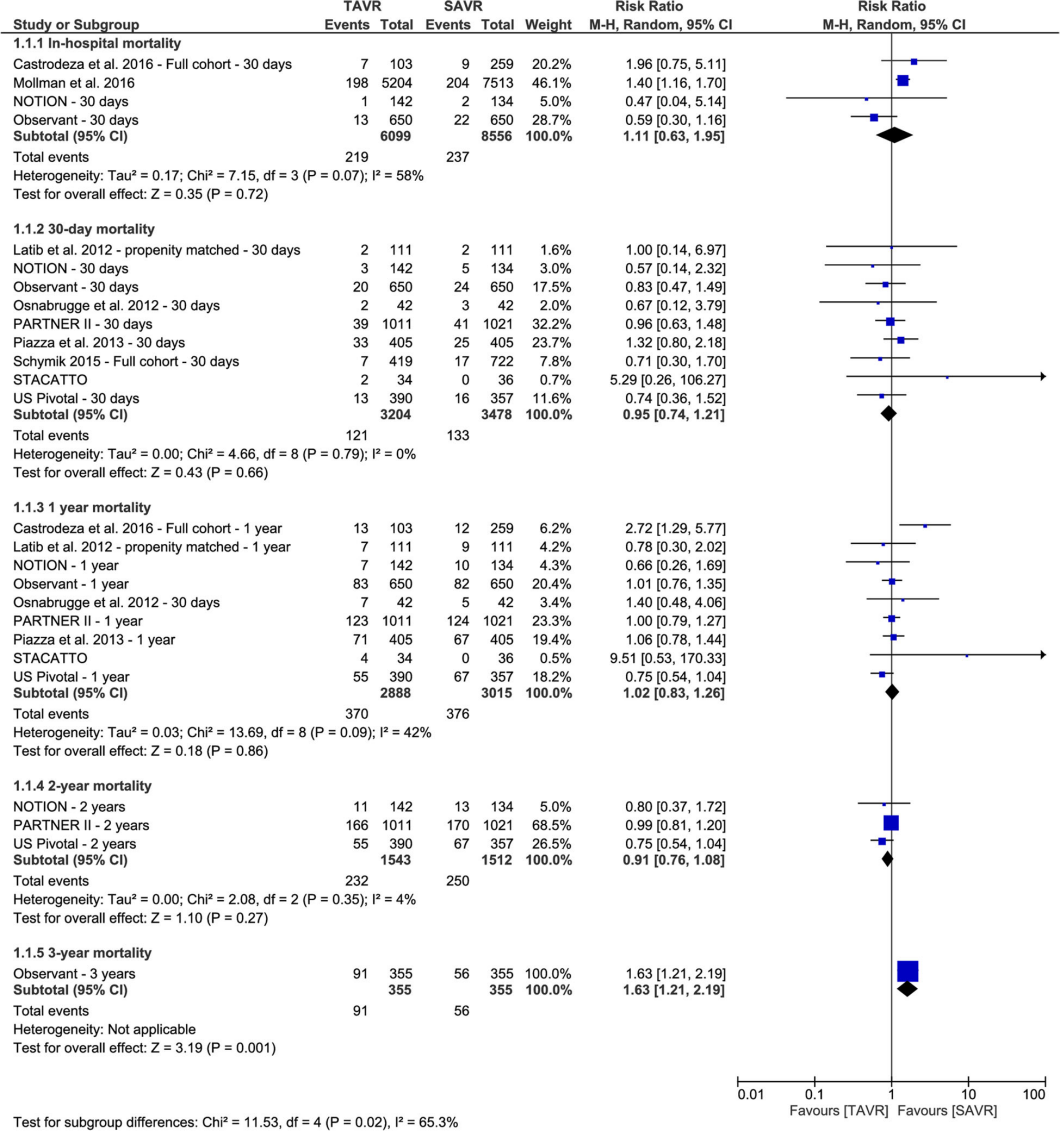
Forest plot of risk ratio of all-cause mortality

The overall RR did not favor either of the two groups in terms of in-hospital mortality (RR 1.11, 95% CI [0.63 to 1.95]), 30-day morality (RR 0.95, 95% CI [0.74 to 1.21]), 1-year mortality (RR 1.02, 95% CI [0.83 to 1.26]), or 2-year mortality (RR 0.91, 95% CI [0.76 to 1.08]). These findings were consistent with another scenario in which we considered pooling of data from RCTs only. The RR of 3-year mortality was reported only by the OBSERVENT study, which showed a significantly higher risk of mortality in the TAVR group than the SAVR group (RR 1.63, 95% CI [1.21 to 2.19]) (Fig. 2).

#### MACCE

**Fig. 3:**
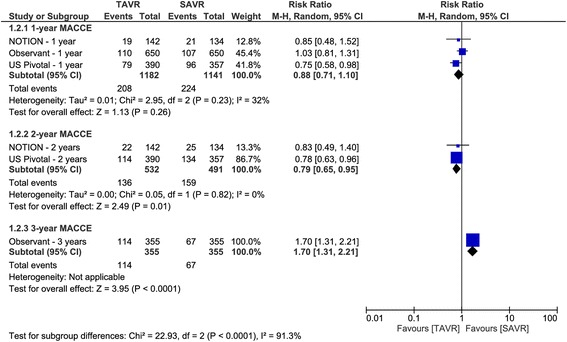
Forest plot of risk ratio of major adverse cardiovascular and cerebrovascular events

The pooled analysis of two RCTs, reporting 1- and 2-year MACCE, favored the TAVR group over the SAVR group (1-year MACCE: RR 0.77, 95% CI [0.61 to 0.98]; and 2-year MACCE: RR 0.79, 95% CI [0.65 to 0.95]). The 30-day MACCE was reported by the US pivotal study only, which showed comparable rates of MACCE between the two groups (RR 0.74, 95% CI [0.47 to 1.18]). Similarly, the 3-year MACCE was reported only by the OBSERVENT study, which favored the SAVR over TAVR (RR 1.70, 95% CI [1.31 to 2.21]) in this regard (Fig. 3).

#### Stroke

**Fig. 4:**
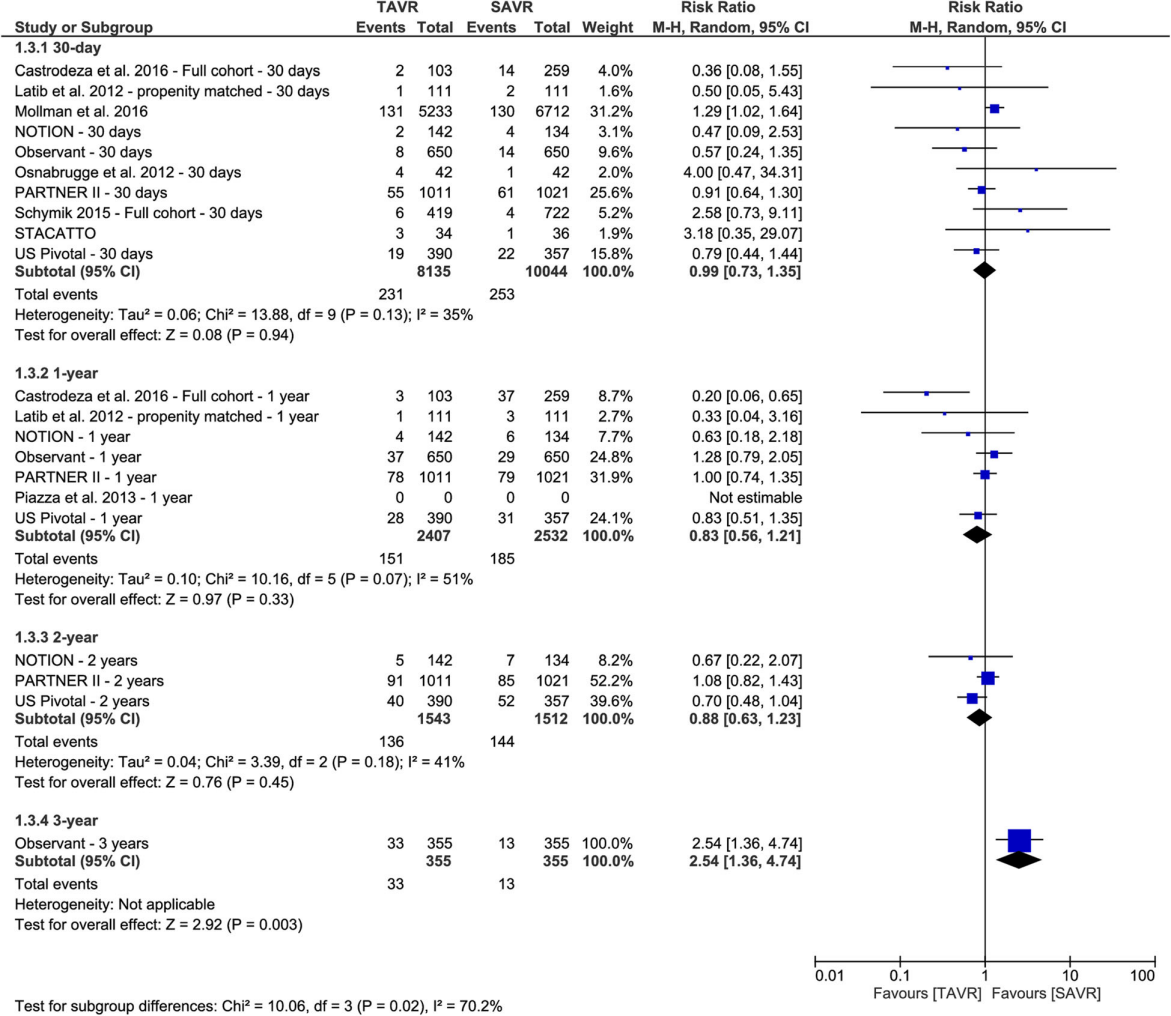
Forest plot of risk ratio of stroke

The overall RR did not favor either of the two groups in terms of stroke incidence within 30 days (RR 0.99, 95% CI [0.73 to 1.35]), 1 year (RR 0.83, 95% CI [0.56 to 1.21]), or 2 years (RR 0.88, 95% CI [0.63 to 1.23]) after the procedure. The OBSERVENT study reported a higher 3-year risk of stroke in the TAVR group (RR 2.54, 95% CI [1.36 to 4.74]), compared to SAVR group. For the risk of stroke at 30 days, there was no evidence of publication bias (*p* = 0.66) (Fig. 4).

#### Myocardial infarction

The overall RR did not favor either of the two groups in terms of myocardial infarction rate within 30 days (RR 0.64, 95% CI [0.39 to 1.04]), 1 year (RR 0.82, 95% CI [0.57 to 1.19]), 2 years (RR 0.96, 95% CI [0.52 to 1.76]), or 3 years (RR 1.20, 95% CI [0.37 to 3.90]). These findings were consistent when we considered pooling data from RCTs only.

#### Major life threatening bleeding

The RR of major bleeding was heterogeneous across studies for 30-day and 1- and 2-year endpoints (I^2^ = 96%, I^2^ = 97%, and I^2^ = 97%, respectively). Out of the 9 studies reporting the risk of major bleeding, seven studies (*n* = 4864 patients) reported a lower incidence of major bleeding in the TAVR group compared with the SAVR group. The STACCATO study showed comparable risk of bleeding between the two groups (1/34 vs. 1/36, respectively), while the ninth study (OBSERVENT) showed a relatively higher incidence of major bleeding in the TAVR group than the SAVR group. When considering data from RCTs only, the pooled RR supported that TAVR has a significantly lower risk of major bleeding than SAVR after 30 days (RR 0.44, 95% CI [0.22 to 0.92]), 1 year (RR 0.45, 95% CI [0.24 to 0.86]), and 2 years (RR 0.48, 95% CI [0.27 to 0.88]).

#### Acute kidney injury

The overall RR of AKI was lower in the TAVR group than the SAVR group at the endpoints of 30 days (RR 0.45, 95% CI [0.34 to 0.59]), 1 year (RR 0.52, 95% CI [0.30 to 0.88]), and 2 years (RR 0.52, 95% CI [0.33 to 0.80]). These findings were consistent with the other scenario in which we considered pooling of data from RCTs only.

#### Vascular access complications

The overall RR showed a higher risk of VAC in the TAVR group compared to the SAVR group at the endpoints of 30 days (RR 12.38, 95% CI [2.46 to 62.28]), 1 year (RR 1.99, 95% CI [1.04 to 3.80]), and 2 years (RR 2.16, 95% CI [1.00 to 4.67]). These findings were consistent when we considered pooling data from RCTs only.

#### Paravalvular aortic regurgitation

The RR of paravalvular AR at 30 days showed a higher incidence of AR in the TAVR group than the SAVR group (RR 3.90, 95% CI [1.25 to 12.12]). Similar results were obtained when pooling data of RCTs only with stratification of AR into mild vs. moderate/severe. The subtotal effect estimates were as follows: (Mild AR: RR 10.21, 95% CI [5.76 to 18.09]; and Moderate/Severe AR: RR 9.30, 95% CI [3.14 to 27.58]).

#### Permanent pacemaker implantation

Compared to SAVR, the risk of PPI was higher in the TAVR group at 30 days (RR 3.31, 95% CI [2.05 to 5.35]), 1 year (RR 2.57, 95% CI [1.36 to 4.86]), but not after 2 years (RR 1.57, 95% CI [0.91 to 2.70]), probably due to the small number of included studies at the 2-year endpoint. When analyzing data from RCTs only, the effect estimate favored the SAVR group over the TAVR group at all endpoints (30-day and one- and two-years).

#### Hospital stay

Six studies reported the duration of hospital stay. Of them, 4 studies showed significantly less hospital stay after TAVR, compared to SAVR. However, the fifth study showed the reverse and the sixth study did not favor either of the two groups. The pooled effect size of hospital stay did not favor either of the two groups (SMD -0.04, 95% CI [−0.34 to 0.26]). However, as we mentioned, this effect size was heterogeneous (I^2^ = 95%).

## Discussion

Since its introduction in 2002, TAVR has attracted the interest of interventional cardiologists as a possible alternative to SAVR [26]. Recently, the clinical practice guidelines of the European Society of Cardiology (ESC) and American College of Cardiology /American Heart Association (ACC/AHA) recommended TAVR as the procedure of choice in high surgical risk patients [27, 28]. However, its value is still debated in AS patients with a low-to-moderate surgical risk. Recently, the ACC added TAVR as a grade IIa recommendation in AS patients with an intermediate surgical risk [29].

Our analysis of data from over 20,000 low-to-moderate risk patients showed no significant difference between SAVR and TAVR in terms of the incidence of all-cause mortality, myocardial infarction, stroke, and MACCE, as well as the length of hospital stay. A higher risk of life-threatening bleeding and AKI was detected in the SAVR group, while the TAVR procedure was associated with a higher risk of VAC and paravalvular AR.

The increased risk of paravalvular AR with TAVR was noted in most included studies, as well as our meta-analysis. This finding can be attributed to multiple valvular and procedural factors, including native valve calcification, the angle of the left ventricle outflow tract to the proximal ascending aorta, inadequate balloon expansion, mismatch between the size of the aortic annulus and the TAVR device, and inadequate deployment technique [30]. However, the procedural limitations are expected to improve with the introduction of newer generation devices and increased TAVR experience among interventional practitioners [31]. Of note, several included trials used the 2-Dimensional echocardiography for valve sizing, which has been shown to cause systematic valve undersizing, leading to a higher incidence of AR, compared to multislice computed tomography (MSCT) [32]. Using MSCT to measure the valve size and the degree of native valve complications can reduce the risk of annulus rupture, coronary artery obstruction, and conduction abnormalities [33, 34].

In the 10 studies that compared both procedures regarding the incidence of stroke, neurological examination was clinically-based and imaging was only requested in patients with evident neurological manifestations. Because the main nuerological outcome was the occurrence of stroke, these studies did not assess for more subtle neurological sypmtoms. In a study by Rodes-Cabau et al., cerebral defects were detected in 70% of patients post-TAVR on magnetic resonance imaging [35]. Future studies should consider incorporating cognitive tests and neuroimaging techniques in their regular neurological evaluations.

As expected, our analysis showed a higher incidence of VAC in the TAVR group, compared to the SAVR group. This finding is commonly explained by the percutaneous nature of the procedure and using large-bore introducer sheaths. In a study by Mussardo et al. [36], a 60% reduction in the incidence of VAC was recorded following the introduction of catheter systems with smaller sheaths, such as the SAPIEN XT and the CoreValve systems. The vascular complications are expected to decrease with the continous improvement of catheter systems.

Although not assessed in this analysis, the PARTNER II trial [8] and SAPIEN III study [37] compared the echocardiographic findings in patients who underwent surgery or transcatheter replacement after 30 days of the procedure. These studies showed that both procedures significantly increased the left ventricular ejection fraction and decreased the mean aortic-valve gradients; however, the improvement was greater in the TAVR group at all time endpoints (up to 2 years following the procedure).

All included studies consistently used the logistic EuroSCORE or the STS risk models for operative risk calculation. However, these score are currently considered outdated and may overestimate the true individual risk [38]. Moreover, it does not consider several elderly-related risk factors, such as fraility, porcelain aorta, malnutrition, and chest deformities [39]. The transcatheter approach may be a better alternative for these patients because they may not be surgical candidates. However, this needs confirmation in future RCTs.

Our results are in accordance with a former meta-analysis of five clinical studies (3199 patients) by Kondur et al. [31]. However, they showed a comparable rate of AKI between both procedures. These differences can be attributed to the marked difference in study inclusion criteria. We believe our results are more credible because they are based on pooling a higher number of clinical trials, as well as observational studies. The low risk of bias in the majority of included studies adds to the credibility of our evidence. Additionally, we performed a subgroup analysis according to the time endpoint at which the outcome was measured (Up to 3 years).

Despite these strength points, our analysis is not without limitations. Observational studies are prone to the effect of unmeasured confounders, which may influence the accuracy of our results. Moreover, meta-analysis of relatively rare events, such as myocardial infarction after these procedures, has its limitations because the occurrence of few events can change the summary effect estimate [40]. Only one study (OBSERVENT) compared both techniques in terms of mortality rate at 3 years and showed a higher risk in the TAVR group; however, more data are needed to confirm this finding.

Future trials are advised to compare the durability of the implanted valves and surgical bioprostheses. Longer follow up periods would be of value because TAVR is likely to expand to younger patients with lower mortality risks. Osnabrugge et al. (2012) compared SAVR and TAVR techniques in terms of the procedural time and costs. Their analysis found a shorter procedural time and a higher cost in the TAVR group, compared to the SAVR group, mostly due to the use of more expensive TAVR devices. These costs were not compensated by the shorter hospital stay and reduced need for blood transfusion in the TAVR group according to their analysis [21].

## Conclusion

In conclusion, our study shows comparable rates of mortality, stroke, and myocardial infarction between SAVR and TAVR groups and a lower risk of life-threatening complications (major bleeding and AKI) in the TAVR group. Although the risks of paravalvular AR and VAC were higher in the TAVR group, these complications are expected to decrease with the continous improvement of catheter systems and TAVR experience among interventional cardiologists. Therefore, TAVR can be an acceptable alternative to SAVR in low-to-moderate risk patients with AS. Larger trials with longer follow-up periods are required to compare the long-term outcomes of both techniques.

## Additional file

Additional file 1: shows risk of bias (ROB) assessment results for included randomized trials and observational studies, according to Cochrane ROB tool and Newcastle-Ottawa Scale. (DOCX 21 kb)

## Abbreviations

AR: Aortic regurgitation
AS: Aortic stenosis
MAACE: Major adverse cardiovascular and cerebrovascular events
SAVR: Surgical aortic valve replacement
TAVR: Transcatheter aortic valve replacement
