# Supplementary material for: Efficacy and safety of transcatheter aortic valve replacement in aortic stenosis patients at low to moderate surgical risk: a comprehensive meta-analysis
Source: BMC Cardiovasc Disord. 2017 Aug 24;17:234. doi: 10.1186/s12872-017-0668-1 (PMC5571502; doi:10.1186/s12872-017-0668-1)
Supplement: Additional file 1: — shows risk of bias (ROB) assessment results for included randomized trials and observational studies, according to Cochrane ROB tool and Newcastle-Ottawa Scale. (DOCX 21 kb) [file 12872_2017_668_MOESM1_ESM.docx]

**Supplementary file 1**

**A. Risk of bias assessment for included randomized controlled trials.**

| **Reason/Quotation** | **Risk of bias** | **PARTNER II 2016** |
| --- | --- | --- |
| "Patients were stratified in cohorts according to access route (transfemoral or transthoracic) and were then randomly assigned (in a 1:1 ratio) to undergo either transcatheter or surgical aortic-valve replacement."  "All the patients were reviewed before randomization by means of teleconference calls by the case-review committee" | Low risk | Random sequence generation (selection bias) |
|  | Low risk | Allocation concealment (selection bias) |
| Blinding is not possible due to the obviously different nature of both procedures. | Low risk | Blinding of participants and personnel (performance bias) |
| Blinding is not possible due to the obviously different nature of both procedures. | Low risk | Blinding of outcome assessment (detection bias) |
| Missing outcome data balanced in numbers across intervention groups, with similar reasons for missing data across groups. | Low risk | Incomplete outcome data (attrition bias) |
| Study’s pre-specified (primary and secondary) outcomes have been reported in the pre-specified way in the protocol: **ClinicalTrials.gov, number NCT01314313.** | Low risk | Selective reporting (reporting bias) |
|  | Unclear | Other bias |
| **Reason/Quotation** | **Risk of bias** | **STACATTO 2012** |
| The 1:1 randomisation between a-TAVI and SAVR was implemented using the web-based clinical trials support system, “TrialPartner” (PCI Research, Aarhus University Hospital, Skejby, Denmark). TrialPartner permits, with a personal log-in, 24-hour randomisation. Data was entered in the electronic case report form of TrialPartner, a secure server based system with security that exceeds the demands and guidelines by the National Data Protection Agency. | Low risk | Random sequence generation (selection bias) |
|  | Low risk | Allocation concealment (selection bias) |
| Blinding is not possible due to the obviously different nature of both procedures. | Low risk | Blinding of participants and personnel (performance bias) |
| Blinding is not possible due to the obviously different nature of both procedures. | Low risk | Blinding of outcome assessment (detection bias) |
| Incomplete outcome data were adequately addressed by intention-to-treat analysis. | Low risk | Incomplete outcome data (attrition bias) |
| Vascular access complications and permanent pacemaker implantation were not reported. | High risk | Selective reporting (reporting bias) |
|  | Unclear | Other bias |
| **Reason/Quotation** | **Risk of bias** | **Notion 2015** |
| The allocation sequence was arranged in permuted blocks | Low risk | Random sequence generation (selection bias) |
| Block size was unknown to the investigators. | Low risk | Allocation concealment (selection bias) |
| Blinding is not possible due to the obviously different nature of both procedures. | Low risk | Blinding of participants and personnel (performance bias) |
| Blinding is not possible due to the obviously different nature of both procedures. | Low risk | Blinding of outcome assessment (detection bias) |
| Incomplete outcome data were adequately addressed by intention-to-treat analysis. | Low risk | Incomplete outcome data (attrition bias) |
| All-important outcomes were reported. | Low risk | Selective reporting (reporting bias) |
|  | Unclear | Other bias |
| **Reason/Quotation** | **Risk of bias** | **US pivotal 2014** |
| Randomization was stratified according to investigational site and intended access site (iliofemoral or noniliofemoral) to ensure proportional assignment. | Low risk | Random sequence generation (selection bias) |
| "Insufficient information" | Unclear risk | Allocation concealment (selection bias) |
| Blinding is not possible due to the obviously different nature of both procedures. | Low risk | Blinding of participants and personnel (performance bias) |
| Blinding is not possible due to the obviously different nature of both procedures. | Low risk | Blinding of outcome assessment (detection bias) |
| Incomplete outcome data were adequately addressed by intention-to-treat analysis. | Low risk | Incomplete outcome data (attrition bias) |
| All-important outcomes were reported. | Low risk | Selective reporting (reporting bias) |
|  | Unclear | Other bias |

**B. Risk of bias assessment for cohort studies**

| **Möllmann 2016** | **Castrodeza 2016** | **Schymik 2015** | **Piazza 2013** | **D'Errigo 2013** | **Study ID** |
| --- | --- | --- | --- | --- | --- |
|  | | | | | **Selection (Max. 4 *)** |
| * | * | * | * | * | Representativeness of the exposed cohort. |
| * | * | * | * | * | Selection of the non-exposed cohort. |
| * | * | * | * | * | Ascertainment of exposure. |
| * | * | * | * | * | Demonstration that outcome of interest was not present at start of study. |
|  | | | | | **Comparability (Max. 2*)** |
| ** | ** | ** | ** | ** | Comparability of cohorts based on the design or analysis. |
|  | | | | | **Outcome (Max. 5 *)** |
| * | * | * |  | * | Assessment of outcome. |
|  | * | * | * | * | Was follow-up long enough for outcomes to occur? |
|  | * | * |  |  | Adequacy of follow up of cohorts. |
| 7 | 9 | 9 | 7 | 8 | **Total** |

**C. Risk of bias assessment for case control studies**

| **Latib 2012** | **Study ID** |
| --- | --- |
| **Selection (Max. 4 *)** | |
| * | Is the case definition adequate? |
| * | Representativeness of the cases |
| * | Selection of Controls |
| * | Definition of Controls |
| **Comparability (Max. 2*)** | |
| * | Study controls for the most important factor (confounder) |
| * | Study controls for any additional factors (confounders) |
| **Exposure (Max. 3 *)** | |
| * | Ascertainment of exposure |
| * | Same method of ascertainment for cases and controls |
|  | Non-Response rate |
| 8 | **Total** |
